# Asystole following Reintubation during Suspension Laryngoscopy

**DOI:** 10.1155/2012/916306

**Published:** 2012-03-26

**Authors:** Sheryl H. Glassman, Michael S. Green, Melissa Brodsky

**Affiliations:** ^1^Drexel University College of Medicine, Hahnemann University Hospital, Philadelphia, PA 19102, USA; ^2^Department of Anesthesiology, Hahnemann University Hospital, Philadelphia, PA 19102, USA

## Abstract

Transient increase in heart rate and mean arterial pressure commonly occur during manipulation of the airway via direct laryngoscopy. This phenomenon is understood to be due to a sympathetic nervous system reflex causing an increase in plasma catecholamines. Rarely, severe bradycardia and possible asystole can occur following laryngoscopy. One previous report described asystole during suspension laryngoscopy after uneventful direct laryngoscopy. Here we report a case of asystole occurring at the time of reinsertion and cuff inflation of an endotracheal tube in a patient who had been hemodynamically stable during initial direct laryngoscopy and the ensuing suspension laryngoscopy. The asystole was immediately recognized and successful cardiopulmonary resuscitation was performed with the patient returning to baseline sinus rhythm. Cardiac arrest following laryngoscopy is rare. This case highlights the importance of continued vigilance even after the initial manipulations of the airway by both direct laryngoscopy and suspension laryngoscopy are to be performed. Identifying patients who may benefit from premedication with a vagolytic drug may prevent adversity. Preoperative heart rate analysis can identify patients with strong vagal tone.

## 1. Introduction

Transient increase in heart rate and mean arterial pressure commonly occur during manipulation of the airway via direct laryngoscopy. This phenomenon is understood to be due to a sympathetic nervous system reflex causing an increase in plasma catecholamines [1]. Rarely, severe bradycardia and possible asystole can occur following laryngoscopy [2]. One previous report described asystole during suspension laryngoscopy after uneventful direct laryngoscopy [2]. This response has been attributed to direct activation of afferent parasympathetic nerve fibers, use of vagotonic opioid drugs, light anesthesia, and hypoxia-induced bradyarrhythmia [3]. We report a case of asystole occurring at time of reinsertion and cuff inflation of an endotracheal tube in a hemodynamically stable patient during direct laryngoscopy and the ensuing suspension laryngoscopy.

## 2. Case Report

 A 56-year-old, 1.92 m, 93 kg male with symptoms of chronic hoarseness and asthma presented for microdirect laryngoscopy with excision of vocal fold masses. His past medical history was significant for juvenile laryngeal papillomatosis. Surgical history consisted of prior excision of multiple vocal cord papillomas and tonsillectomy. Standard ASA monitoring was employed. Prior to induction the blood pressure was 147/92 and electrocardiogram showed sinus rhythm at 64 beats per minute. Respiratory rate was 16 and pulse oximetry showed 100% on room air. Airway exam revealed Mallampati class III and the thyromental distance and mouth opening within normal limits.

Preoxygenation with 100% oxygen preceded induction with propofol 200 mg and fentanyl 100 mcg. Muscle relaxation with rocuronium 50 mg facilitated intubation. A Macintosh 3 blade revealed a Cormack and Lehane grade II view. A 6.5 endotracheal tube was passed atraumatically and uneventfully. Anesthesia was maintained with desflurane (1.2 minimum alveolar concentration) and oxygen (FiO_2_ of 0.96). The operating table was rotated 90 degrees and the patient was placed in suspension by the otolaryngologist. Additional doses of rocuronium maintained paralysis and fentanyl boluses provided analgesia.

 One hour into the procedure, difficulty in ventilation was noted. The surgeon revealed that the endotracheal tube cuff had been punctured. With the patient still in suspension, the existing endotracheal tube was removed and a new 6.5 cuffed endotracheal tube inserted. Within one minute sinus bradycardia ensued. Glycopyrrolate 0.2 mg was immediately given intravenously.

The patient's heart rate continued to rapidly decrease to eventual asystole. Atropine 1 mg was administered intravenously. Exam revealed no palpable radial pulse. The patient was taken out of suspension and cardiopulmonary resuscitation was initiated per ACLS protocol for approximately five minutes. Asystole converted to sinus tachycardia.

Postcardiac arrest electrocardiogram, arterial blood gas, chemistry panel, and cardiac enzymes were obtained and within normal limits. Sustained tetanus with 50 Hz for 5.0 sec via neuromuscular twitch monitor preceded muscle relaxant reversal with glycopyrrolate 0.8 mg and to neostigmine 0.05 mg/kg. The patient was awakened and extubated uneventfully.

## 3. Discussion

 Cardiac arrest following intubation is rare, especially intraoperatively following endotracheal tube exchange [4]. On thorough review of this case, it is believed that the asystole occurred secondary to several contributions: strong vagal reflex, suspension laryngoscopy, vagotonic drugs (fentanyl), and reinsertion of the endotracheal tube causing stimulation of the larynx and trachea.

 A strong vagal reflex is more commonly seen in the pediatric population which has implemented the use of atropine as a premedication [5]. The belief is that during laryngoscopy, an arrest of the sinoatrial node occurs with a simultaneous impulse via the atrioventricular node leading to bradycardia and asystole. This patient displayed strong vagal tone throughout the case. Patients intubated under suspension laryngoscopy can have a more pronounced vagal response to the stimulation than direct laryngoscopy. When utilizing suspension, as opposed to the Macintosh curved blade, the undersurface of the epiglottis is stimulated. This portion of the airway is innervated by the internal branch of the superior laryngeal nerve derived from the vagus nerve. Stimulation of this can promote bradycardia.

 Identifying patients who may benefit from premedication with a vagolytic drug such as atropine can prevent adversity. Preoperative heart rate analysis can identify patients with strong vagal tone. Patients intubated via suspension laryngoscopy may warrant prophylactic vagolytics. The threshold for administration of a vagolytic while a patient is in suspension should be lower especially if the surgeon is operating on the glottis. This case highlights the importance of continued vigilance even following the initial manipulation of the airway by both direct laryngoscopy and suspension laryngoscopy.
